# A Metric on the Space of *k*th-order reduced Phylogenetic Networks

**DOI:** 10.1038/s41598-017-03363-y

**Published:** 2017-06-09

**Authors:** Juan Wang, Maozu Guo

**Affiliations:** 1 School of Computer Science, Inner Mongolia University, Hohhot, 010021 P.R. China; 2 School of Electrical and Information Engineering, Beijing University of Civil Engineering and Architecture, Beijing, 100044 P.R. China

## Abstract

Phylogenetic networks can be used to describe the evolutionary history of species which experience a certain number of reticulate events, and represent conflicts in phylogenetic trees that may be due to inadequacies of the evolutionary model used in the construction of the trees. Measuring the dissimilarity between two phylogenetic networks is at the heart of our understanding of the evolutionary history of species. This paper proposes a new metric, i.e. *k*th-distance, for the space of *k*th-order reduced phylogenetic networks that can be calculated in polynomial time in the size of the compared networks.

## Introduction

Phylogenetic networks play a vital role in the description of the evolutionary history of species, and are especially appropriate for datasets whose evolutions contain significant amounts of reticulate events caused by recombination, hybridization, horizontal gene transfer, gene duplication, gene conversion and loss^1–7^. Even for the species which have evolved based on a tree-like model of evolution, phylogenetic networks can be used to represent conflicts in phylogenetic trees that may be caused by inadequacies of an used evolutionary model. So far, there have been many algorithms and programs for constructing phylogenetic networks. The assessment of the algorithms for constructing phylogenetic networks is mainly by means of the comparison of the networks, for example, comparing the constructed network with simulate network or actual network. In addition, comparing two phylogenetic networks can help us to understand the evolutionary history of species. Recently, researchers have shown an increased interest in definition of metrics for computing the dissimilarity between a pair of phylogenetic networks.

A measure *d* is called a metric on a space *S* if it satisfies four properties: for any *a*, *b*, *c* ∈ *S*:

- *d*(*a*, *b*) ≥ 0 (nonnegative);
- *d*(*a*, *b*) = 0 if and only if *a* = *b* (i.e. *a* and *b* are isomorphic) (reflexivity);
- *d*(*a*, *b*) = *d*(*b*, *a*) (symmetry);
- *d*(*a*, *b*) + *d*(*b*, *c*) ≥ *d*(*a*, *c*) (triangle inequality).

In general, it is much easier to prove a defined measure to satisfy the above-mentioned properties except the reflexivity. For a metric, if two phylogenetic networks are isomorphic, the distance between them computed by the metric is 0, otherwise it is 1; then we say that the metric is trivial. A trivial metric satisfies obviously above-mentioned properties, but it doesn’t show other information about evolutionary history implied by the two phylogenetic networks. Accordingly, in addition to these four properties, it is desired that the metric can give us some information on the dissimilarity of the evolutionary histories expressed by the phylogenetic networks being compared^8–13^.

Up to now, several metrics have been designed and proven that each one of them is a metric on a certain subspace of rooted phylogenetic networks, for example, *μ*-metric on the space of tree-sibling phylogenetic networks^14^, the tripartition metric on the space of tree-child phylogenetic networks^15–18^, the *m*-distance on the space of reduced phylogenetic networks^19^, and the *d*_*e*_-distance on the space of partly reduced phylogenetic networks^20^. The largest one among those subspace is the partly reduced phylogenetic networks, so the *d*_*e*_-distance is also the metric on the subspaces of tree-child phylogenetic networks, tree-sibling phylogenetic networks and reduced phylogenetic networks. The paper will introduce a new metric, denoted by *k*th-distance, on space of *k*th-order reduced phylogenetic networks (will be discussed in the following sections), and the metric is polynomial-time computable. The space of *k*th-order reduced phylogenetic networks is larger subspace of rooted phylogenetic networks than any one subspace on which has been defined a metric. If no special instructions, the rest of paper will use the network to denote the rooted phylogenetic network.

## Preliminaries

Let ${\mathscr{X}}$ be a set of taxa. A rooted phylogenetic network *N* = (*V*, *E*) on ${\mathscr{X}}$ is a directed acyclic graph (DAG for short), with one root node, and its leaves labelled as ${\mathscr{X}}$ by a bijection *f*.

For a network *N* = (*V*, *E*) and a node *u* ∈ *V*, if:

- indeg(*u*) = 0, then *u* is the root;
- indeg(*u*) ≤ 1, then *u* is a tree node;
- indeg(*u*) ≥ 2, then *u* is a reticulate node;
- outdeg(*u*) = 0, then *u* is a leaf;
- outdeg(*u*) ≥ 1, then *u* is an internal node.

Sometimes we use the notation *N* = ((*V*, *E*), *f*) to denote the network *N*, and *V*_*N*_ to denote the leaf set of *N*. Given two nodes *u*, *v* ∈ *V*. If (*u*, *v*) ∈ *E*, then we say that *v* is a child of *u* or *u* is a parent of *v*. If there exists a directed path from *u* to *v*, then we say that *v* is a descendant of *u* or *u* is an ancestor of *v*.

The height of a node *u* is the length of a longest directed path beginning from *u* and ending with a leaf. The non-existence of cycles indicates that all nodes of *N* can be categorized by height: the nodes with height 0 are the leaves; for a node *u* with height *a* > 0, each child of *u* has height *m* < *a* and there exists at least one child with height exactly *a* − 1.

The depth of a node *v* is the length of a longest directed path beginning from the root and ending with *v*. In the same way, the non-existence of cycles indicates that all nodes of *N* can be categorized by depth: the only node with depth 0 is the root; for a node *v* with depth *b* > 0, each parent of *v* has depth *m* < *b* and there exists at least one parent with depth exactly *b* − 1.

**Definition 1**. For two networks *N*_1_ = ((*V*_1_, *E*_1_), *f*_1_) and *N*_2_ = ((*V*_2_, *E*_2_), *f*_2_), they are isomorphic if and only if there exists a bijection *H* from *V*_1_ to *V*_2_ such that:

- (*u*, *v*) is an edge in *E*_1_ if and only if (*H*(*u*), *H*(*v*)) is an edge in *E*_2_;
- for each leaf *w* ∈ *V*_1_, *f*_1_(*w*) = *f*_2_(*H*(*w*)).

**Figure 1:**
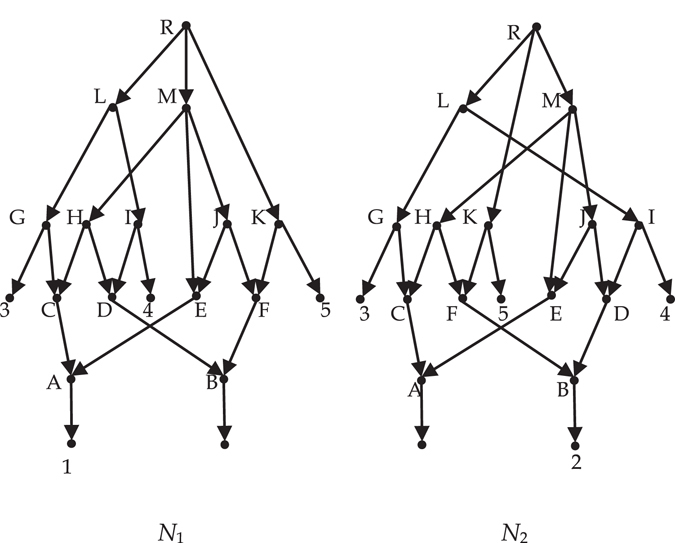
*N*_1_ and *N*_2_ are not isomorphic.

**Figure 2:**
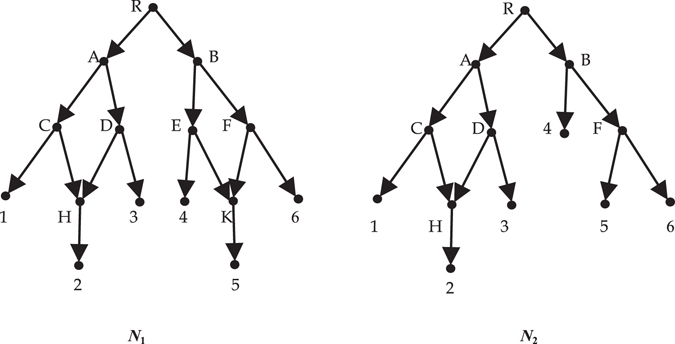
*N*_1_ and *N*_2_ on ${\mathscr{X}}=\{1,2,3,4,5,6\}$ are not isomorphic.

**Figure 3:**
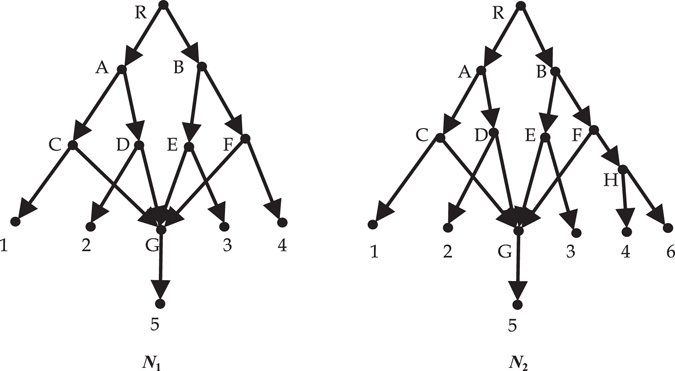
*N*_1_ is on the ${{\mathscr{X}}}_{1}=\{1,2,3,4,5\}$; *N*_2_ is on the ${{\mathscr{X}}}_{2}=\{1,2,3,4,5,6\}$.

Although the subspace defined by the *d*_*e*_-distance is the largest one among all defined subspaces, there exist a large number of networks that aren’t measured by the *d*_*e*_-distance. For example, the two networks in Fig. 1 (from the paper^20^) are not isomorphic, while the *d*_*e*_-distance between them is 0. Even for two non-isomorphic networks whose *d*_*e*_-distance is not 0, the distance is usually maximal value 1. For example the networks in Fig. 2, there is a certain resemblance between them, so it is desired that the distance between them is less than 1. However, their *d*_*e*_-distance is maximal value 1. On the other hand, for any two networks *N*_1_ on ${{\mathscr{X}}}_{1}$ and *N*_2_ on ${{\mathscr{X}}}_{2}$, the *d*_*e*_-distance between them is 1 as long as ${{\mathscr{X}}}_{1}\ne {{\mathscr{X}}}_{2}$. When ${{\mathscr{X}}}_{1}\subset {{\mathscr{X}}}_{2}$, the two compared networks may share some information (see Fig. 3).

## Methods

Let *N* = ((*V*, *E*), *f*) be a network. Now we begin to give several definitions for the same network.

**Definition 2**. Two nodes *u*, *v* ∈ *V* (not necessarily different) are called first-order equivalent, denoted by *u* ≡ ^1^ *v*, if

- *u*, *v* ∈ *V*_*N*_ and *f*(*u*) = *f*(*v*), or
- node *u* has *l*(≥1) children ${u}_{1},{u}_{2},\cdots ,{u}_{l}$, node *v* has *l* children ${v}_{1},{v}_{2},\cdots ,{v}_{l}$, and *u*_*i*_ ≡ ^1^ *v*_*i*_ for 1 ≤ *i* ≤ *l*.

**Example 1**. Consider the network *N*_1_ in Fig. 1. Each node of *N*_1_ is first-order equivalent with itself, and C ≡ ^1^E, D ≡ ^1^F, H ≡ ^1^J.

**Definition 3**. Given an even number *k* ≥ 2. Two nodes *u*, *v* ∈ *V* (not necessarily different) are called *k*th-order equivalent, denoted by *u* ≡ ^*k*^ *v*, if *u* ≡ ^*k*−1^ *v*, and:

- *u*, *v* are the root, or
- node *u* has *l*(≥1) parents ${u}_{1},{u}_{2},\cdots ,{u}_{l}$, node *v* has *l* parents ${v}_{1},{v}_{2},\cdots ,{v}_{l}$, and *u*_*i*_ ≡ ^*k*^ *v*_*i*_ for 1 ≤ *i* ≤ *l*.

**Definition 4**. Given an odd number *k* ≥ 2. Two nodes *u*, *v* ∈ *V* (not necessarily different) are called *k*th-order equivalent, denoted by *u* ≡ ^*k*^ *v*, if *u* ≡ ^*k*−1^ *v*, and:

- *u*, *v* ∈ *V*_*N*_, and *f*(*u*) = *f*(*v*), or
- node *u* has *l*(≥1) children ${u}_{1},{u}_{2},\cdots ,{u}_{l}$, node *v* has *l* children ${v}_{1},{v}_{2},\cdots ,{v}_{l}$, and *u*_*i*_ ≡ ^*k*^ *v*_*i*_ for 1 ≤ *i* ≤ *l*.

**Example 2**. Consider the network *N*_1_ in Fig. 1 again. Each node of *N*_1_ is second-order equivalent with itself, and H ≡ ^2^J. Each node of *N*_1_ is only *k*th-order equivalent with itself (*k* ≥ 3).

**Figure 4:**
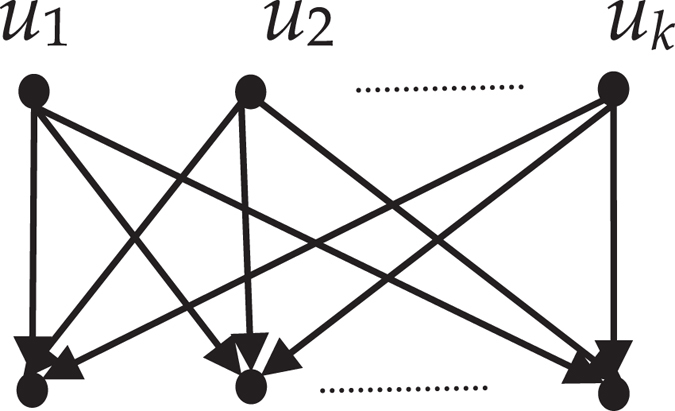
The topology relation of odd-order equivalent nodes.

**Lemma 1**. *Here k is an odd number. Given nodes u*_1_, *u*_2_, $\cdots$, *u*_*s*_ *in a network, if each u*_*i*_ *has l children, and each child of u*_*i*_ *is only k*th*-order equivalent with itself (*1 ≤ *i* ≤ *s). Then u*_1_ ≡ ^*k*^ *u*_2_ ≡ ^*k*^ $\cdots$  ≡ ^*k*^ *u*_*s*_ *if and only if u*_1_, *u*_2_, $\cdots$, *u*_*s*_ *have the same children (refer to the* Fig. 4 *)*.

**Figure 5:**
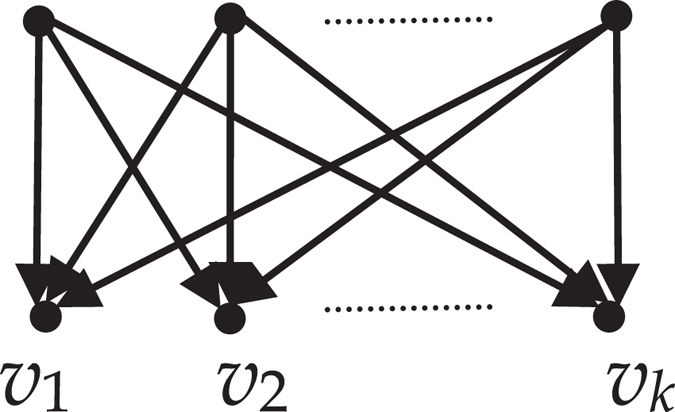
The topology relation of even-order equivalent nodes.

**Lemma 2**. *Here k is an even number. Given nodes v*_1_, *v*_2_, $\cdots$, *v*_*s*_ *in a network, if each v*_*i*_ *has l parents, and each parent of v*_*i*_ *is only k*th*-order equivalent with itself. Then v*_1_ ≡ ^*k*^ *v*_2_ ≡ ^*k*^ $\cdots$ ≡ ^*k*^ *v*_*s*_ *if and only if v*_1_, *v*_2_, $\cdots$, *v*_*s*_ *have the same parents (refer to the* Fig. 5 *)*.

**Lemma 3**. *For all leaves, the root and the nodes with height 1 in a network, each of them is k*th*-order equivalent with itself (for any k)*.

The proofs of Lemmas 1, 2 and 3 aren’t listed here. It can be concluded from these definitions that each *k*th-order equivalence is an equivalence relation, i.e. it is transitive, reflexive and symmetric. It can be easily proved that all the first-order equivalent nodes have the same height and all the *k*th-order equivalent nodes (*k* ≥ 2) have the same height and depth (refer to the literature^20^).

If a node *u* is *k*th-order equivalent with other nodes except itself, we say that *u* has non-trivial *k*th-order equivalent nodes. For a network, after deleting the non-trivial *k*th-order equivalent nodes of each node, as well as the nodes with indegree 1 and outdegree 1, the resulting network is called the *k*th-order reduced phylogenetic network. All the *k*th-order reduced phylogenetic networks form the space of *k*th-order reduced phylogenetic network. So a network *N* is in the space of *k*th-order reduced phylogenetic networks, if and only if each node of *N* is only *k*th-order equivalent with itself.

**Figure 6:**
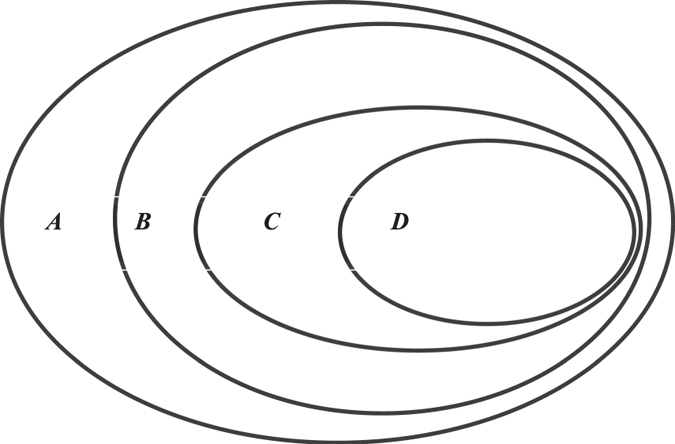
*A* is the space of rooted phylogenetic networks; *B* is the space of *k*th-order reduced phylogenetic networks (*k* ≥ 2); *C* is the space of partly reduced phylogenetic networks; and *D* is the space of reduced phylogenetic networks.

The space of first-order reduced phylogenetic networks is the space of reduced phylogenetic networks defined in the paper^19^. The space of second-order reduced phylogenetic networks is the space of partly reduced phylogenetic networks defined in the paper^20^. Figure 6 shows the relationship of these subspaces.

**Figure 7:**
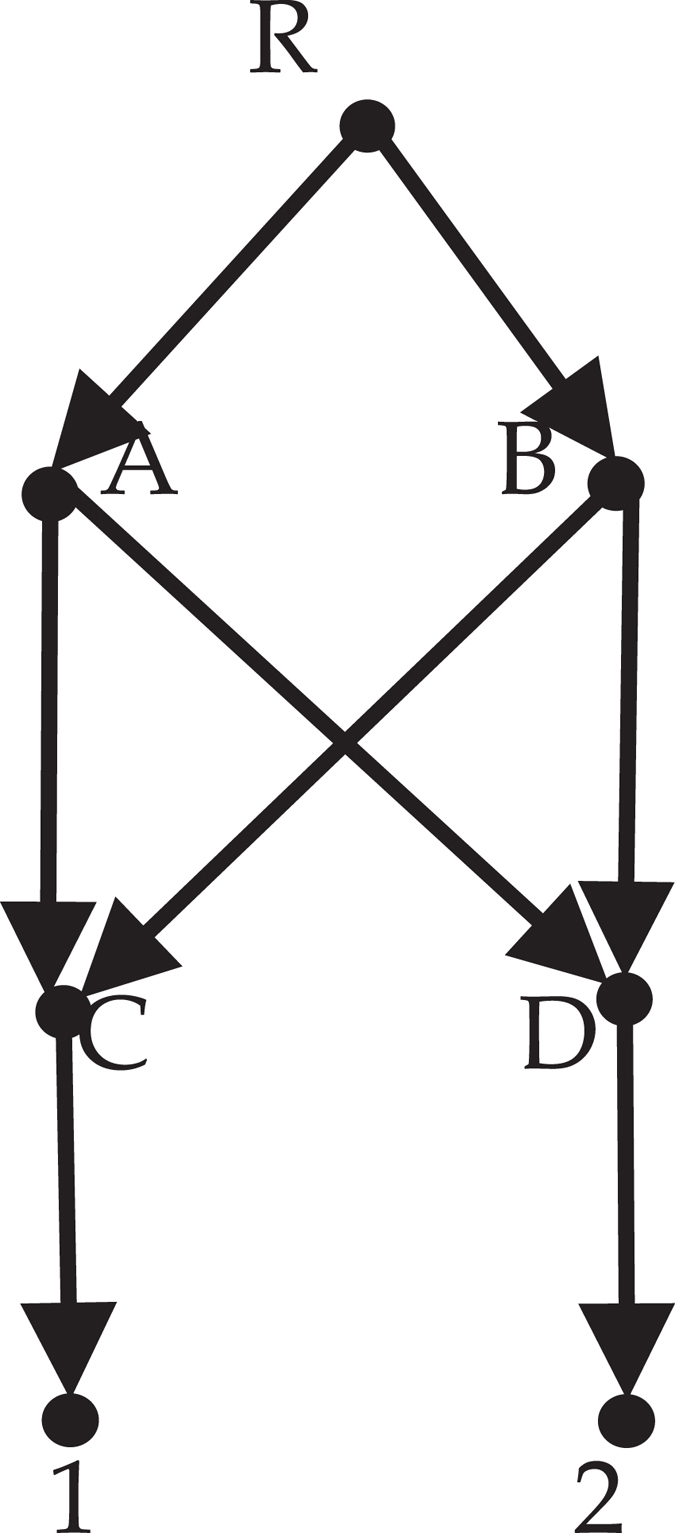
*N* is a rooted phylogenetic network.

The space of *k*th-order reduced phylogenetic networks is not equals to the space of rooted phylogenetic network. For example the network *N* in Fig. 7, for any *k*, each node of *N* is *k*th-order equivalent with itself, and A ≡ ^*k*^B. So *N* isn’t the *k*th-order reduced phylogenetic network, i.e. not in the space of *k*th-order reduced phylogenetic networks.

In order to compute the dissimilarity of the networks, we will extend the above concepts defined in a network to two networks in the following sections. Let *N*_1_ = ((*V*_1_, *E*_1_), *f*_1_) and *N*_2_ = ((*V*_2_, *E*_2_), *f*_2_) be two networks.

**Definition 5**. Two nodes *u* ∈ *V*_1_, *v* ∈ *V*_2_ are called first-order equivalent, denoted by *u* ≡ ^1^ *v*, if

- $$u\in {V}_{{N}_{1}},v\in {V}_{{N}_{2}}$$, and *f*_1_(*u*) = *f*_2_(*v*), or
- node *u* has *l*(≥1) children *u*_1_, *u*_2_, $\cdots$, *u*_*l*_, node *v* has *l* children *v*_1_, *v*_2_, $\cdots$, *v*_*l*_, and *u*_*i*_ ≡ ^1^ *v*_*i*_ for 1 ≤ *i* ≤ *l*.

**Definition 6**. Given an even number *k* ≥ 2. Two nodes *u* ∈ *V*_1_, *v* ∈ *V*_2_ are called *k*th-order equivalent, denoted by *u* ≡ ^*k*^ *v*, if *u* ≡ ^*k*−1^ *v*, and:

- *u*, *v* are the root, or
- node *u* has *l*(≥1) parents *u*_1_, *u*_2_, $\cdots$, *u*_*l*_, node *v* has *l* parents *v*_1_, *v*_2_, $\cdots$, *v*_*l*_, and *u*_*i*_ ≡ ^*k*^ *v*_*i*_ for 1 ≤ *i* ≤ *l*.

**Definition 7**. Given an odd number *k* ≥ 2. Two nodes *u* ∈ *V*_1_, *v* ∈ *V*_2_ are called *k*th-order equivalent, denoted by *u* ≡ ^*k*^ *v*, if *u* ≡ ^*k*−1^ *v*, and:

- $$u\in {V}_{{N}_{1}},v\in {V}_{{N}_{2}}$$ and *f*_1_(*u*) = *f*_2_(*v*), or
- node *u* has *l*(≥1) children *u*_1_, *u*_2_, $\cdots$, *u*_*l*_, node *v* has *l* children *v*_1_, *v*_2_, $\cdots$, *v*_*l*_, and *u*_*i*_ ≡ ^*k*^ *v*_*i*_ for 1 ≤ *i* ≤ *l*.

Let *u*, *u*_0_ be two nodes from two networks or the same network. From these definitions, it follows that if there exists a positive integer *k*_1_, such that *u* ≢ ${}^{{k}_{1}}{u}_{0}$, then for any *k* > *k*_1_, *u* ≢ ^k^ *u*_0_. Given two networks *N*_1_ = (*V*_1_, *E*_1_) and *N*_2_ = (*V*_2_, *E*_2_). We use the following processes to compute the *k*th-order unique nodes of *N*_1_, denoted by *L* ^*k*^(*N*_1_). First *L* ^*k*^(*N*_1_) = ∅. Then for each node *u* ∈ *V*_1_, if there has no node *u*_0_ ∈ *L* ^*k*^(*N*_1_) such that *u* ≡ ^*k*^ *u*_0_, add *u* to *L* ^*k*^(*N*_1_). Similarly, we can compute *L* ^*k*^(*N*_2_). For each node *u* ∈ *L* ^*k*^(*N*_1_), ${e}_{{N}_{1}}^{k}(u)$ denotes the number of nodes which are *k*th-order equivalent with *u*, i.e. ${e}_{{N}_{1}}^{k}(u)=|\{v\in {V}_{1}:v{\equiv }^{k}u\}|$. Similarly, we can define ${e}_{{N}_{2}}^{k}(u)$ for each node *u* ∈ *L* ^*k*^(*N*_2_). For the sake of simplicity, we drop the subscript of *e*. Here *e* ^*k*^(∅) = 0.

**Lemma 4**. *Given two networks N*_1_ = (*V*_1_, *E*_1_) *and N*_2_ = (*V*_2_, *E*_2_)*. For u*_1_, *u*_2_ ∈ *V*_1_ *, v*_1_, *v*_2_ ∈ *V*_2_ *, and u*_1_ ≡ ^*k*^ *v*_1_, *u*_2_ ≡ ^*k*^ *v*_2_ *. Then, u*_1_ ≡ ^*k*^ *u*_2_ *if and only if v*_1_ ≡ ^*k*^ *v*_2_.

*Proof*. Refer to the proof of the Theorem 15 in the paper^20^.◽

## A Metric

**Definition 8**. For two networks *N*_1_ = (*V*_1_, *E*_1_) and *N*_2_ = (*V*_2_, *E*_2_), the *k*th-distance *d*_*k*_(*N*_1_, *N*_2_) equals

1 $$\frac{1}{k({n}_{1}+{n}_{2})}\{\sum _{i=1}^{k}[\sum _{v\in {L}^{i}({N}_{1})}max\{0,{e}^{i}(v)-{e}^{i}(v^{\prime} )\}+\sum _{u\in {L}^{i}({N}_{2})}max\{0,{e}^{i}(u)-{e}^{i}(u^{\prime} )\}]\}$$

where *v*′ (or *u*′) is a node in *L* ^*i*^(*N*_2_) (or *L* ^*i*^(*N*_1_)) that is *i*th-order equivalent to *v* (or *u*), and if no such node exists, then *v*′ = ∅ (or *u*′ = ∅). *n*_1_ and *n*_2_ are the number of nodes in *N*_1_ and *N*_2_ respectively.

For each *i* (1 ≤ *i* ≤ *k*), the maximal value of ${\sum }_{v\in {L}^{i}({N}_{1})}max\{\mathrm{0,}\,{e}^{i}(v)-{e}^{i}(v^{\prime} )\}+{\sum }_{u\in {L}^{i}({N}_{2})}max\{\mathrm{0,}\,{e}^{i}(u)-{e}^{i}(u^{\prime} )\}$ is *n*_1_ + *n*_2_, so the formulate 1 has maximal value 1 and minimal value 0. For a give *i* (1 ≤ *i* ≤ *k*), if the value of ${\sum }_{v\in {L}^{i}({N}_{1})}max\{\mathrm{0,}\,{e}^{i}(v)-{e}^{i}(v^{\prime} )\}+{\sum }_{u\in {L}^{i}({N}_{2})}max\{\mathrm{0,}\,{e}^{i}(u)-{e}^{i}(u^{\prime} )\}$ is *d*, then for any *j* (*i* + 1 ≤ *j* ≤ *k*), the value of ${\sum }_{v\in {L}^{j}({N}_{1})}max\{\mathrm{0,}\,{e}^{j}(v)-{e}^{j}(v^{\prime} )\}+{\sum }_{u\in {L}^{j}({N}_{2})}max\{\mathrm{0,}\,{e}^{j}(u)-{e}^{j}(u^{\prime} )\}$ is more than *d*.

From the definition 8, it follows that the 1st-distance is the *m*-distance defined in the space of reduced phylogenetic networks, and the 2nd-distance is the *d*_*e*_-distance defined in the space of partly reduced phylogenetic networks.

**Lemma 5**. *If d*_*k*_(*N*_1_, *N*_2_) = 0*. Then* |*V*_1_| = |*V*_2_|*, and there exists a node v*_0_ ∈ *L* ^*i*^(*V*_2_) *for each node v* ∈ *L* ^*i*^(*V*_1_)*, such that v*_0_ ≡ ^*i*^ *v and e* ^*i*^(*v*_0_) = *e* ^*i*^(*v*) *(*1 ≤ *i* ≤ *k)*.

*Proof*. From *d*_*k*_(*N*_1_, *N*_2_) = 0, it follows that ${\sum }_{v\in {L}^{i}({N}_{1})}max\{0,{e}^{i}(v)-{e}^{i}(v^{\prime} )\}=0$ and ${\sum }_{u\in {L}^{i}({N}_{2})}max\{\mathrm{0,}{e}^{i}(u)-{e}^{i}(u^{\prime} )\}=0$ (1 ≤ *i* ≤ *k*). So *max*{0, *e* ^*i*^(*v*) − *e* ^*i*^(*v*′)} = 0 for each node *v* ∈ *L* ^*i*^(*N*_1_). Suppose that there exists a node *v* ∈ *L* ^*i*^(*N*_1_) such that *e* ^*i*^(*v*) − *e* ^*i*^(*v*′) < 0, then *e* ^*i*^(*v*′) − *e* ^*i*^(*v*) > 0. So ${\sum }_{u\in {L}^{i}({N}_{2})}max\{0,{e}^{i}(u)-{e}^{i}(u^{\prime} )\} > 0$. It contradict ${\sum }_{u\in {L}^{i}({N}_{2})}max\{0,{e}^{i}(u)-{e}^{i}(u^{\prime} )\}=0$. Therefore, for each node *v* ∈ *L* ^*i*^(*N*_1_), we have *e* ^*i*^(*v*) − *e* ^*i*^(*v*′) = 0, i.e. *e* ^*i*^(*v*) = *e* ^*i*^(*v*′). Similarly, for each node *u* ∈ *L* ^*i*^(*N*_2_), *e* ^*i*^(*u*) = *e* ^*i*^(*u*′). Accordingly, |*V*_1_| = |*V*_2_|.◽

**Lemma 6**. *Given two k*th*-order reduced phylogenetic networks N*_1_ = (*V*_1_, *E*_1_) *and N*_2_ = (*V*_2_, *E*_2_)*. Then d*_*k*_(*N*_1_, *N*_2_) = 0 *if and only if N*_1_ *and N*_2_ *are isomorphic*.

*Proof*. If *N*_1_ and *N*_2_ are isomorphic, obviously *d*_*k*_(*N*_1_, *N*_2_) = 0. The converse conclusion will be proven as follows.

Lemma 5 tells us that |*V*_1_| = |*V*_2_|. From the property of the *k*th-order reduced phylogenetic networks, it follows that each node *u* in *V*_1_ is just *k*th-order equivalent with itself and *u* ∈ *L* ^*k*^(*V*_1_). Similarly, each node *v* in *V*_2_ is just *k*th-order equivalent with itself and *v* ∈ *L* ^*k*^(*V*_2_). Moreover, for each node *u* ∈ *V*_1_, there exists the only one node *v* ∈ *V*_2_ such that *u* ≡ ^*k*^ *v*. So we define a mapping *H* from *V*_1_ to *V*_2_, for each node *u* ∈ *V*_1_, *H*(*u*) = *u*′, where *u*′ ∈ *V*_2_ and *u*′ ≡ ^*k*^ *u*.

First we prove that the mapping *H* is a bijection. For any two different nodes *u*_1_, *u*_2_ ∈ *V*_1_, there exist two nodes ${u}_{1}^{^{\prime} },{u}_{2}^{^{\prime} }\in {V}_{2}$, such that $H({u}_{1})={u}_{1}^{^{\prime} }$ and $H({u}_{2})={u}_{2}^{^{\prime} }$. Here ${u}_{1}^{^{\prime} }$ and ${u}_{2}^{^{\prime} }$ are not the same nodes. If not, then *u*_1_ ≡ ^*k*^ *u*_2_. It contradict that each node *u* ∈ *V*_1_ is just *k*th-order equivalent with itself. So *H* is injective. Due to |*V*_1_| = |*V*_2_|, we have that *H* is a surjection.

Then we prove that if (*u*, *v*) ∈ *E*_1_, then (*H*(*u*), *H*(*v*)) ∈ *E*_2_. Let *u*_0_ = *H*(*u*) and *v*_0_ = *H*(*v*), i.e. *u*_0_ ≡ ^*k*^ *u* and *v*_0_ ≡ ^*k*^ *v*. If *k* is an odd number, then the children of *u* are *k*th-order equivalent with the children of *u*_0_ respectively. Thus, *v* is *k*th-order equivalent with a child *v*′ of *u*_0_, i.e. *v*′ ≡ ^*k*^ *v* ≡ ^*k*^ *v*_0_. Since every node is only *k*th-order equivalent with itself, *v*′ and *v*_0_ are the same nodes, i.e. *v*_0_ is a child of *u*_0_. Therefore, (*u*_0_, *v*_0_) ∈ *E*_2_. Similarly, we can come to the conclusion when *k* is an even number.

The mapping *H* also preserves the labels of the leaves from the definition of *k*th-order equivalence. In conclusion, *N*_1_ and *N*_2_ are isomorphic.

**Lemma 7**. *For any one pair of networks N*_1_ *and N*_2_, *d*_*k*_(*N*_1_, *N*_2_) = *d*_*k*_(*N*_2_, *N*_1_).

The distance *d*_*k*_(*N*_1_, *N*_2_) can be viewed as the symmetric difference of the same set of elements ${\cup }_{i=1}^{k}\{{L}^{i}({N}_{1})\cup {L}^{i}({N}_{2})\}$. From the property of the symmetric difference^21^, it follows that the following triangle inequality holds:

**Lemma 8**. *For any three networks N*_1_ *, N*_2_ *and N*_3_ *, d*_*k*_(*N*_1_, *N*_2_) + *d*_*k*_(*N*_2_, *N*_3_) ≥ *d*_*k*_(*N*_1_, *N*_3_).

From Lemmas 6, 7 and 8, we have the following result:

**Theorem 9** *The k*th*-distance defined by the formula 1 is a metric on the space of k*th*-order reduced phylogenetic networks*.

Let *k* = 3 and *n*_*j*_ the number of nodes of network *N*_*j*_ (*j* = 1, 2). Consider the two networks in Fig. 1. For *i* = 1 and 2, ${\sum }_{v\in {L}^{i}({N}_{1})}max\{0,{e}^{i}(v)-{e}^{i}(v^{\prime} )\}+{\sum }_{u\in {L}^{i}({N}_{2})}max\{0,{e}^{i}(u)-{e}^{i}(u^{\prime} )\}=0$. For *i* = 3, ${\sum }_{v\in {L}^{i}({N}_{1})}max\{0,{e}^{i}(v)-{e}^{i}(v^{\prime} )\}+{\sum }_{u\in {L}^{i}({N}_{2})}max\{0,{e}^{i}(u)-{e}^{i}(u^{\prime} )\}={n}_{1}+{n}_{2}$. So the *d*(*N*_1_, *N*_2_) = 1/3.

Consider two networks in Fig. 2. The nodes *R*, *B*, *E*, *F*, *K* in *V*_1_ don’t exist first-order equivalent nodes in *V*_2_, while the nodes *R*, *B*, *F* in *V*_2_ don’t exist first-order equivalent nodes in *V*_1_. Everyone else has only one first-order equivalent node. So ${\sum }_{v\in {L}^{1}({N}_{1})}max\{0,{e}^{1}(v)-{e}^{1}(v^{\prime} )\}+{\sum }_{u\in {L}^{1}({N}_{2})}max\{0,{e}^{1}(u)-{e}^{1}(u^{\prime} )\}=8$. For *i* = 2 and 3, every node in *V*_1_ doesn’t exist *i*th-order equivalent nodes in *V*_2_. So ${\sum }_{v\in {L}^{i}({N}_{1})}max\{0,{e}^{i}(v)-{e}^{i}(v^{\prime} )\}+{\sum }_{u\in {L}^{i}({N}_{2})}max\{0,{e}^{i}(u)-{e}^{i}(u^{\prime} )\}$ $={n}_{1}+{n}_{2}=13+15=28$. Accordingly *d*(*N*_1_, *N*_2_) = (8 + 28 + 28)/(3 × 28) = 16/21.

Consider two networks in Fig. 3. The nodes *R*, *B*, *F* in *V*_1_ don’t exist first-order equivalent nodes in *V*_2_, and the nodes *R*, *B*, *F*, *H*, 6 in *V*_2_ don’t exist first-order equivalent nodes in *V*_1_. Everyone else has only one first-order equivalent with node. So ${\sum }_{v\in {L}^{1}({N}_{1})}max\{0,{e}^{1}(v)-{e}^{1}(v^{\prime} )\}+{\sum }_{u\in {L}^{1}({N}_{2})}max\{0,{e}^{1}(u)-{e}^{1}(u^{\prime} )\}=8$. For *i* = 2 and 3, every node in *V*_1_ doesn’t exist *i*th-order equivalent nodes in *V*_2_. So ${\sum }_{v\in {L}^{i}({N}_{1})}max\{0,{e}^{i}(v)-{e}^{i}(v^{\prime} )\}+{\sum }_{u\in {L}^{i}({N}_{2})}max\{0,{e}^{i}(u)-{e}^{i}(u^{\prime} )\}$ $={n}_{1}+{n}_{2}=13+15=28$. Accordingly *d*(*N*_1_, *N*_2_) = (8 + 28 + 28)/(3 × 28) = 16/21.

**Lemma 10**. *If there is d*_*k*_(*N*_1_, *N*_2_) = 0 *for all k. Then there exists a positive integer m, such that for any m*_0_ ≥ *m, we have that each node u in V*_1_ *has a m*_0_ *th-order equivalent node u*′ *in V*_2_.

*Proof*. Assume that the above conclusion does not hold, i.e. for any positive integer *m*, there exist *k*_0_ ≥ *m* and a node *u* ∈ *V*_1_, such that *u*′ ≢ ${}^{{k}_{0}}u$ for any node *u*′ ∈ *V*_2_. So when *m* = 1, there exist *k*_1_ and *u*_1_ ∈ *V*_1_, such that *u*_1_ ≢ ${}^{{k}_{1}}u^{\prime}$ for any node *u*′ ∈ *V*_2_. So ${d}_{{k}_{1}}({N}_{1},{N}_{2})\ne 0$. This conclusion is in contradiction with *d*_*k*_(*N*_1_, *N*_2_) = 0 for all *k*.   ◽

### Computational Aspects

For odd number *k* (or even number *k*), the *k*th-order equivalent nodes can be computed by a bottom-up (or top-down) approach, no matter whether the nodes are in the same network or two different networks. Given two networks *N*_1_ = ((*V*_1_, *E*_1_), *f*_1_) and *N*_2_ = ((*V*_2_, *E*_2_), *f*_2_). Algorithm 8 shows the pseudocode that decides whether two nodes are *k*th-order equivalent or not, where E(*k*) is the abbreviation for the set of *k*th-order equivalent nodes. This process will cost at most *O*(*n* ^3^) time, where *n* = max(|*V*_1_|, |*V*_2_|). Therefore, it takes totally at most *O*(*n* ^5^) time to find out all *i*th-order (where 1 ≤ *i* ≤ *k*) equivalent nodes for each node of the two networks. Computing the formula 1 will costs *O*(*n*) time. In conclusion, we will spend *O*(*n* ^5^) time in computing the *k*th-distance between two networks, where *n* is the maximum of |*V*_1_| and |*V*_2_|.

## Results

We compared the *k*th-distance with *m*-distance on the space of reduced phylogenetic networks^19^ and the *d*_*e*_-distance on the space of partly reduced phylogenetic networks^20^, by means of 100 networks constructed by the Lnetwork method^3^

**Table:** 

**Algorithm 1**: Deciding whether *u* and *v* are *k*th-order equivalent or not for an odd number *k* (or an even number *k*).
1: input: nodes *u* and *v*
2: **if** outdegree of *u* is not equals to that of *v* (or indegree of *u* is not equals to that of *v*) **then**
3: return
4: **end if**
5: **if** *u* and *v* are leaves and they have the same labels (or *u* and *v* are the root) **then**
6: add *v* to E(*k*) of *u*
7: add *u* to E(*k*) of *v*
8: **else**
9: flag := false
10: **if** E(*k* − 1) of *u* does’t contain *v* **then**
11: return
12: **end if**
13: **for** each child *a* of *u* (or each parent *a* of *u*) **do**
14: **for** each child *b* of *v* (or each parent *b* of *v*) **do**
15: **if** *b*.*label* = true **then**
16: continue
17: **end if**
18: **if** the E(*k*) of *a* has *b* **then**
19: flag = true
20: *b*.*label* = true
21: **end if**
22: **end for**
23: **if** flag = false **then**
24: return
25: **else**
26: flag = false
27: **end if**
28: **end for**
29: add *v* to E(*k*) of *u*
30: add *u* to E(*k*) of *v*
31: **end if**

**Figure 8:**
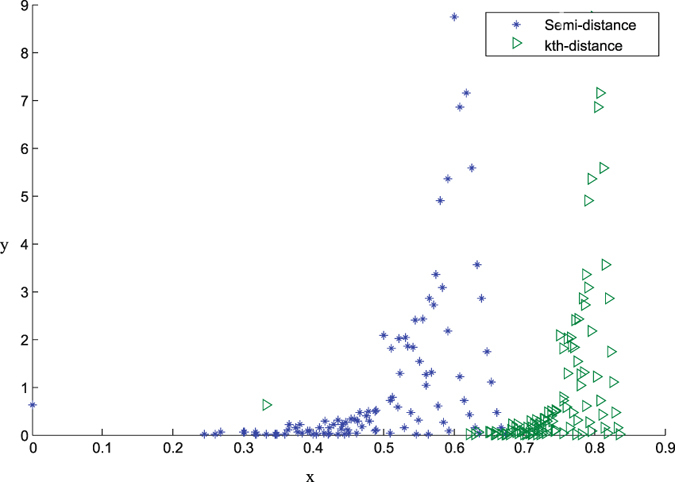
The results of *m*-distance and *k*th-distance.

. Thus, each distance method can obtain a distance matrix with approximately 5000 values. Figure 8 shows the distribution of the distance values, where the horizontal axis is the distance value and the vertical axis is the percent of the distance value in all values. Here the results of *d*_*e*_-distance didn’t show in Fig. 8, because it just has two distance values 1 and 0, and 99.38 percent and 0.62 percent respectively. The minima of *m*-distance and the *d*_*e*_-distance are 0, while the minimum of *k*th-distance is 0.32.

From the results, we reached the following conclusions. First, almost all *d*_*e*_-distance values are maximum values 1. Second, the *k*th-distance values are not 0 between the networks whose *d*_*e*_-distance and the *m*-distance values are 0. Third, the *k*th-distance values are larger than the *m*-distance values for the same networks.

## Discussion

**Figure 9:**
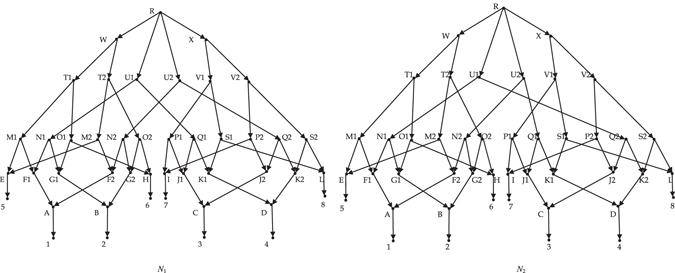
Two networks are not isomorphic.

In order to compare dissimilarity for more phylogenetic networks, we define a polynomial-time computable metric on the space of *k*th-order reduced phylogenetic networks. Here the larger *k* is, the larger the space of *k*th-order reduced phylogenetic networks is. Moreover, the larger *k* is, the more precise the distance between two phylogenetic networks is. Take the non-isomorphism networks in Fig. 1 for example. When *k* = 1 or 2, the value computed by the formula 1 is 0, i.e. their *m*-distance and *d*_*e*_-distance are 0. However, when *k* = 3, the value computed by the formula 1 is 1/3. So when *k* = 1 or 2, the value computed by the formula 1 doesn’t indicate the real dissimilarity between the two networks. The choose of *k* in general is based on the desired precision of distance. Whatever *k* is, the *k*th-distance is not a metric on the space of all rooted phylogenetic networks. For example, the two phylogenetic networks in Fig. 9, their *k*th-distance is 0, but they are not isomorphic.
